# Supplementary material for: Babesia bovis Enolase Is Expressed in Intracellular Merozoites and Contains B-Cell Epitopes That Induce Neutralizing Antibodies In Vitro
Source: Vaccines (Basel). 2025 Jul 31;13(8):818. doi: 10.3390/vaccines13080818 (PMC12389923; doi:10.3390/vaccines13080818)
Supplement: Supplementary file 1 [file vaccines-13-00818-s001.zip › vaccines-3672408-supplementary.pdf]

Supplementary Materials

A)

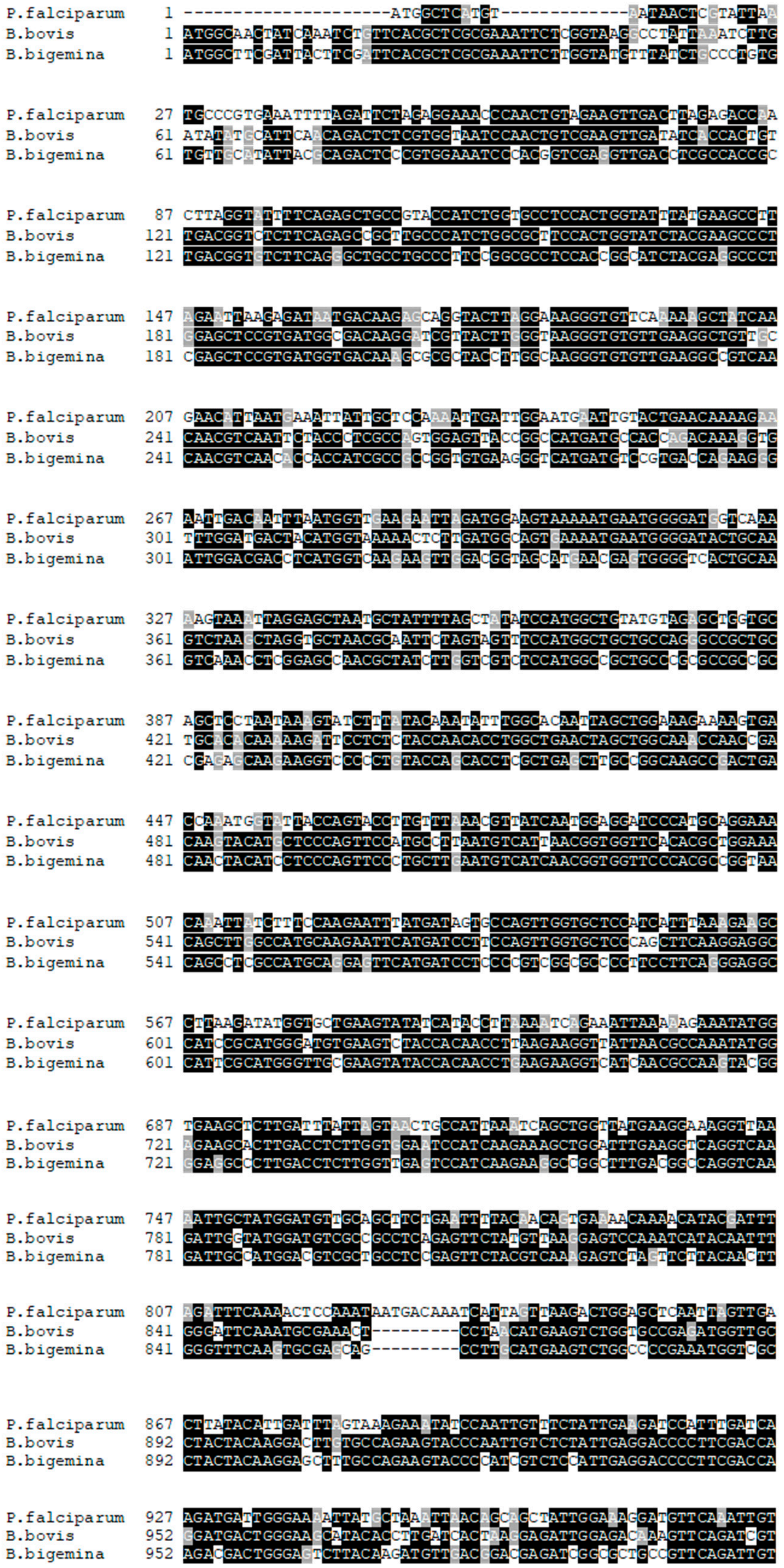

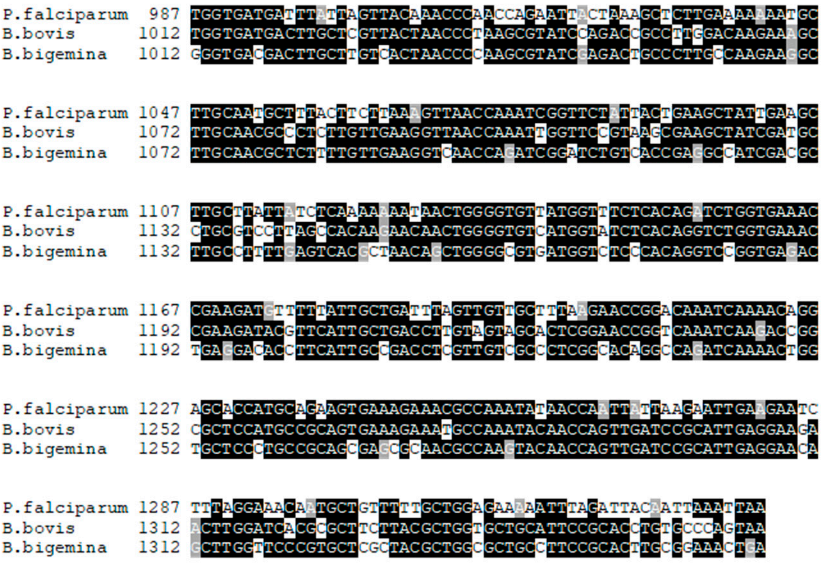

B)

	P. falciparum	*B. bovis*	*B. bigemina*
P. falciparum	100.00	62.76	59.91
B. bovis	62.76	100.00	76.28
B. bigemina	59.91	76.28	100.00

Figure S1. (A) Nucleotide multiple alignment of predicted *B. bovis enolase*, identified in Chromosome III with genomic sequence of T2Bo strain (AAXT01000001.1) with enolases from *P. falciparum* (XM_004221995.1) and *B. bigemina* (MK490919.1) obtained with Clustal W and edited with boxshade. Identical nucleotides are displayed in white letters on a black background, while substitutions are highlighted with a shaded background. (B) percentage identity matrix of referred sequences obtained with Clustal Omega.

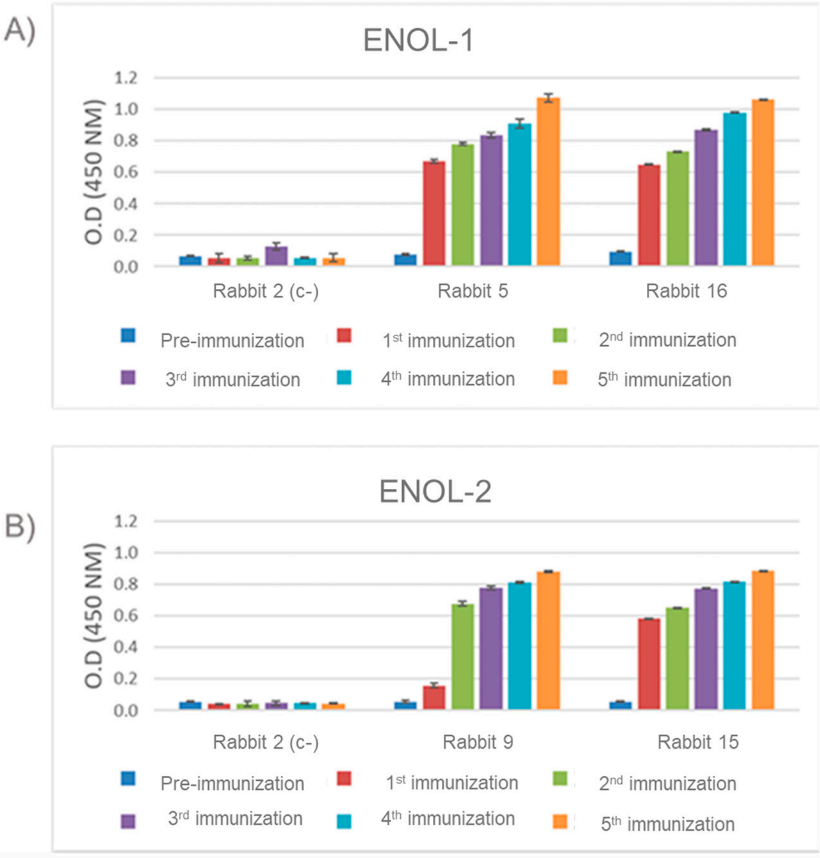

Figure S2. Antibody determination graphs against *B. bovis* enolase peptides. Panels (A,B) show the different antibody levels against the synthetic peptides after each immunization. All sera were used at 1:2000 dilution in PBS.

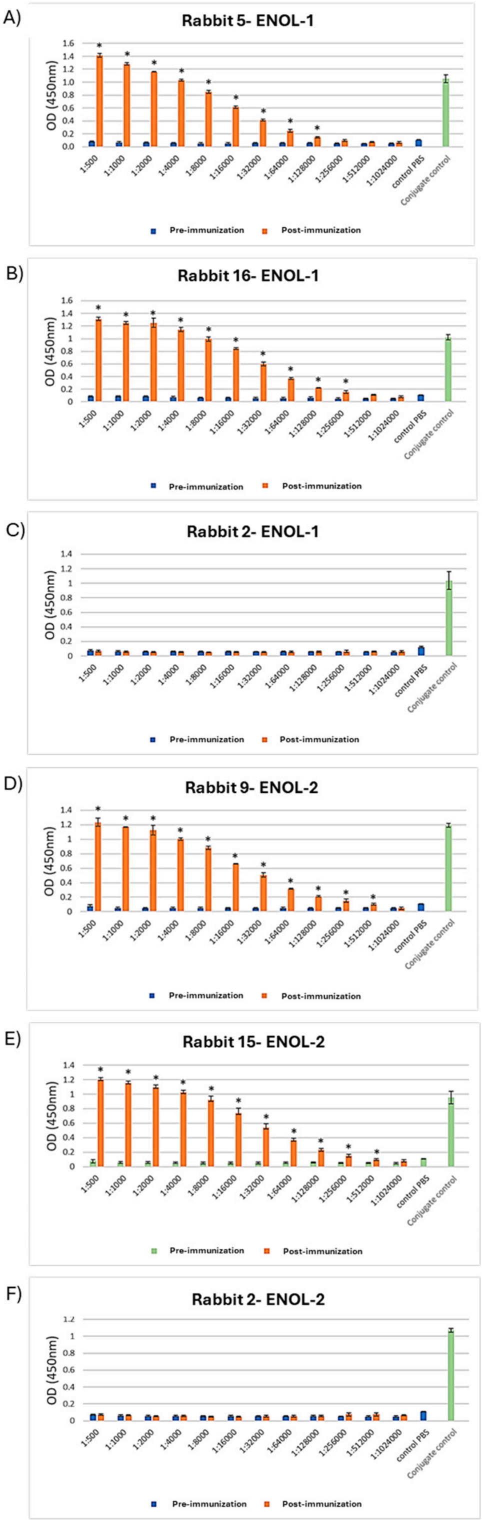

Figure S3. Antibody titration graphs against *B. bovis* enolase peptides. Panels (A–C) show the different dilutions of the serum from rabbits immunized with the ENOL-1 peptide and the control rabbit immunized only with PBS and adjuvant. Panels (D–F) show the different dilutions of the serum from rabbits immunized with the ENOL-2 peptide and the control rabbit immunized only with PBS and adjuvant. Dilutions with a statistically significant difference between the pre-immunization serum and the 5th immunization serum ($p < 0.05$) are indicated with *.
