# Supplementary figures and images for: Babesia bovis Enolase Is Expressed in Intracellular Merozoites and Contains B-Cell Epitopes That Induce Neutralizing Antibodies In Vitro
Source: Vaccines (Basel). 2025 Jul 31;13(8):818. doi: 10.3390/vaccines13080818 (PMC12389923; doi:10.3390/vaccines13080818)

**File S1.** The original Western blot figures.

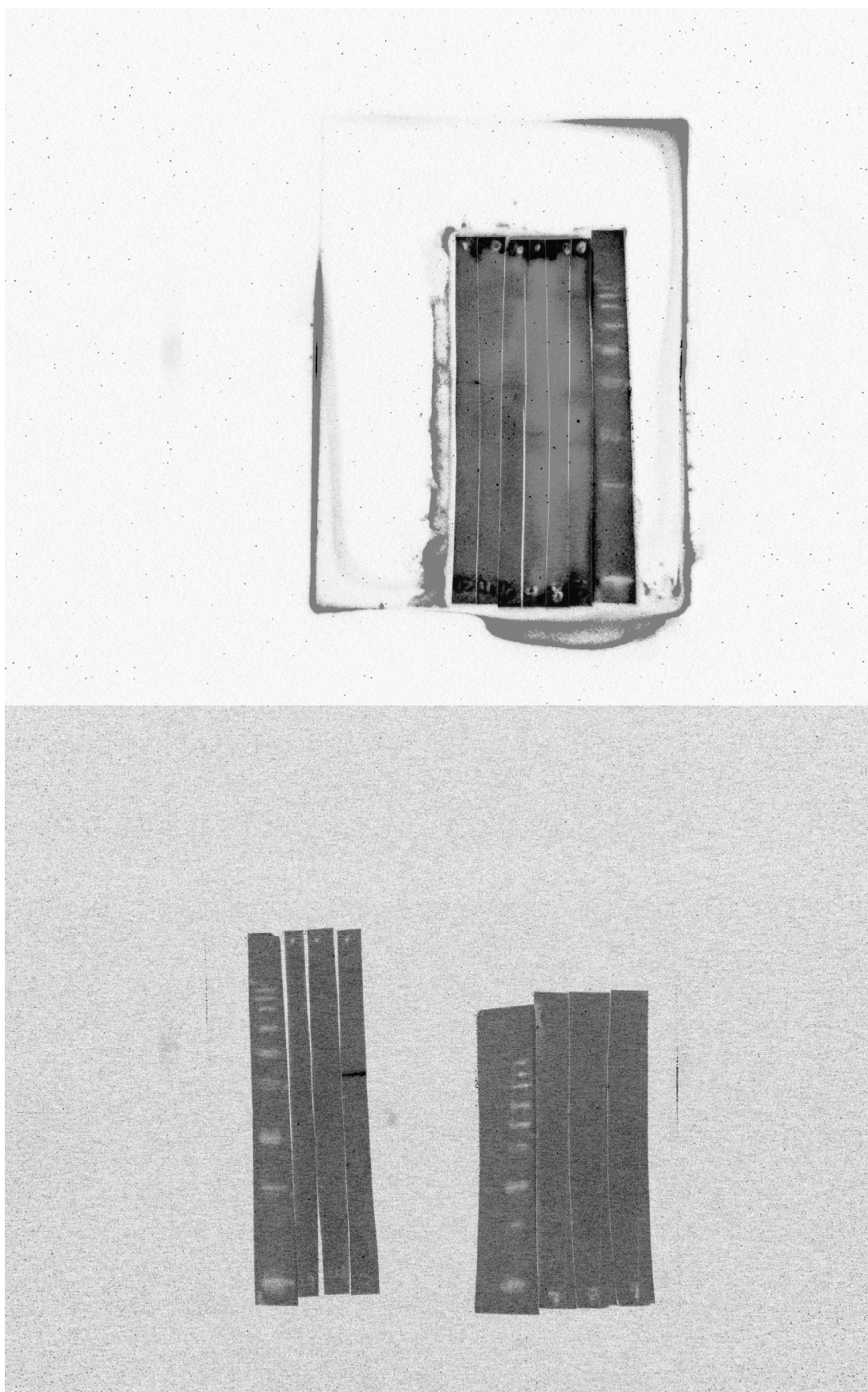

Supplement: Supplementary file 1 [file vaccines-13-00818-s001.zip › vaccines-3672408-File S1.pdf]
